# Management of Psychosis in Perinatal Period in Local Psychiatric Inpatient Unit, Current Pathways and Proposal to Improve Standards of Care

**DOI:** 10.1192/bjo.2025.10341

**Published:** 2025-06-20

**Authors:** Irum Bibi, Oleksandr Khrypunov, Nimisha Agarwal, Kazi Rahman

**Affiliations:** Humber NHS Foundation Trust, Hull, United Kingdom

## Abstract

**Aims:** To enhance the quality of care for patients experiencing psychotic episodes during the perinatal period (antenatal and postnatal) by improving service pathways and management.

**Methods:** This was a retrospective study, in which existing care pathways for patients presenting with psychosis in perinatal period were reviewed, gaps identified and then solutions to improve standards were proposed. Out of 41 patients admitted to the local inpatient unit with psychosis in perinatal period between January 2022 and October 2024, 11 patients were selected as they met the criteria of diagnosis of perinatal psychosis. Data was collected and reviewed from electronic records and patients’ notes.

Data was assessed, whether the key elements for patients presenting with psychosis were documented in admission history, the management plans and the extent of involvement of perinatal team throughout different stages of their care.

**Results:** It was found that 90% of patients (10/11) were clerked on admission. In 63% patients (7/11) reasons of admission were documented, and 54.5% (6/11) having documentation about parity.

81.8% (9 of 11) had perinatal team involvement during admission, 45.45% (5 of 11) had discharge follow up with perinatal team, while 27.27 % (3 of 11) were discharged to other teams. Only 9% (1/11) were asked about perinatal family history, 81% (9/11) were not asked about perinatal family history while 1 patient had missing clerking documentation.

**Conclusion:** There were notable gaps picked up in clerking history especially perinatal family history, gestational age and parity, which are critical points in history taking in patients presenting with psychosis in perinatal period. While there is consistent involvement of perinatal team during admission, there seems to be lack of consistency in post-discharge engagement.

The data suggested more standardised clerking, and discharge planning process to ensure all element of care are covered. By implementing the proposed, we anticipate a positive impact on patient outcome, more cohesive multidisciplinary care and improved patient follow up leading to better quality care.

